# Rebuttal from Francisco J. Arjona and Jeroen H. F. de Baaij

**DOI:** 10.1113/JP275705

**Published:** 2018-01-31

**Authors:** Francisco J. Arjona, Jeroen H. F. de Baaij

**Affiliations:** ^1^ Department of Physiology, Radboud Institute for Molecular Life Sciences Radboud University Medical Center Nijmegen The Netherlands

**Keywords:** CNNM2, CNNM4, cyclin, magnesium, hypomagnesemia

We fully agree with Funato and colleagues on the relevance of CNNM proteins for the maintenance of Mg^2+^ homeostasis (Funato *et al*. 2018). Both of our papers summarize the solid body of evidence demonstrating that CNNM2 is key for renal Mg^2+^ handling, while CNNM4 regulates intestinal Mg^2+^ transport.

Nevertheless, we do not share the conclusions presented by Funato and colleagues suggesting that CNNMs are Na^+^/Mg^2+^ exchangers. Funato and colleagues mainly base their view on the use of the Magnesium‐Green fluorescent probe in an immortalized cell line (HEK293) (Yamazaki *et al*. 2013; Hirata *et al*. 2014). The restrictions of the use fluorescent Mg^2+^ indicators, due to Ca^2+^ sensitivity and high dissociation constants, are widely described (Günther, 2006). Indeed, other groups using fluorescent Mg^2+^ probes could not show CNNM‐induced Mg^2+^ fluxes or even measure Mg^2+^ influx against the Na^+^ gradient (Hardy *et al*. 2015; Sponder *et al*. 2016). Moreover, the Mg^2+^ efflux experiments of Funato and colleagues were reported to be performed by bathing the cells in a solution with 40 mm Mg^2+^, which will certainly surpass physiological intracellular Mg^2+^ concentrations (Yamazaki *et al*. 2013; Hirata *et al*. 2014). In contrast, when CNNM2 is overexpressed in HEK293 cells and Mg^2+^ efflux is measured under physiological conditions with the stable ^25^Mg^2+^ isotope, no Mg^2+^ efflux can be attributed to CNNM2 (Arjona *et al*. 2014).

Furthermore, our group was the first to show CNNM‐mediated Na^+^ influx that can be inhibited by a high extracellular Mg^2+^ concentration in 2011 (Stuiver *et al*. 2011). Based on these experiments we concluded that CNNM2 cannot act as Na^+^/Mg^2+^ exchanger because: (i) the inward Na^+^ current was also present when applying Mg^2+^‐free pipette solution, (ii) the presumed exchanger did not function in the opposite direction, and (iii) the reversal potential that we measured was not in agreement with the theoretical reversal potential of a Na^+^/Mg^2+^ exchanger (Stuiver *et al*. 2011). Funato and colleagues repeated these electrophysiological experiments in similar conditions and found the same results (Yamazaki *et al*. 2013). Despite the discrepancies that we've mentioned, they find support in these experiments to claim Na^+^/Mg^2+^ exchange.

In conclusion, the experimental evidence reported by Funato and colleagues does not support the notion that CNNM proteins are genuine Na^+^/Mg^2+^ exchangers, though they influence renal and intestinal Mg^2+^ transport. In line with our claim that CNNM proteins are not Na^+^/Mg^2+^ exchangers, Funato and colleagues write that: “it still remains uncertain whether they are genuine exchangers by themselves or cooperatively function with one or several further proteins” (Funato *et al*. 2018). In this regard, these authors allude to the need to clarify whether CNNM proteins may interact with the well‐characterized Na^+^/Mg^2+^ exchangers of the SLC41 family (Kolisek *et al*. 2012; de Baaij *et al*. 2016).

## Call for comments

Readers are invited to give their views on this and the accompanying CrossTalk articles in this issue by submitting a brief (250 word) comment. Comments may be submitted up to 6 weeks after publication of the article, at which point the discussion will close and the CrossTalk authors will be invited to submit a ‘LastWord’. Please email your comment, including a title and a declaration of interest, to jphysiol@physoc.org. Comments will be moderated and accepted comments will be published online only as ‘supporting information’ to the original debate articles once discussion has closed.

## Additional information

### Competing interests

None declared.

### Author contributions

Both authors have approved the final version of the manuscript and agree to be accountable for all aspects of the work. All persons designated as authors qualify for authorship, and all those who qualify for authorship are listed.

### Funding

This work was financially supported by grants from the Netherlands Organization for Scientific Research (NWO VENI 016.186.012) and the Dutch Kidney Foundation (Kolff 14OKG17).
